# Genetics of Wellbeing and Its Components Satisfaction with Life, Happiness, and Quality of Life: A Review and Meta-analysis of Heritability Studies

**DOI:** 10.1007/s10519-015-9713-y

**Published:** 2015-02-26

**Authors:** Meike Bartels

**Affiliations:** 1 Department of Biological Psychology, Netherlands Twin Register, VU University Amsterdam, Van der Boechorststraat 1, 1081 BT Amsterdam, The Netherlands; 2 EMGO+ Institute for Health and Care Research, VU University Medical Centre, Amsterdam, The Netherlands; 3 Neuroscience Campus Amsterdam, Amsterdam, The Netherlands

**Keywords:** Wellbeing, Satisfaction with life, Happiness, Twin, Heritability, Review, Meta-analysis, Genetics, Genes

## Abstract

Wellbeing is a major topic of research across several disciplines, reflecting the increasing recognition of its strong value across major domains in life. Previous twin-family studies have revealed that individual differences in wellbeing are accounted for by both genetic as well as environmental factors. A systematic literature search identified 30 twin-family studies on wellbeing or a related measure such as satisfaction with life or happiness. Review of these studies showed considerable variation in heritability estimates (ranging from 0 to 64 %), which makes it difficult to draw firm conclusions regarding the genetic influences on wellbeing. For overall wellbeing twelve heritability estimates, from 10 independent studies, were meta-analyzed by computing a sample size weighted average heritability. Ten heritability estimates, derived from 9 independent samples, were used for the meta-analysis of satisfaction with life. The weighted average heritability of wellbeing, based on a sample size of 55,974 individuals, was 36 % (34–38), while the weighted average heritability for satisfaction with life was 32 % (29–35) (n = 47,750). With this result a more robust estimate of the relative influence of genetic effects on wellbeing is provided.

## Introduction

In recent years, wellbeing has become a topic of research across several scientific disciplines. A major force driving this broad interest is the association of wellbeing with physical and mental health and its possible pivotal role in socio-economic issues and economic development (Boehm et al. 2011; Greenspoon and Saklofske 2010; Seaford 2011; Stiglitz et al. 2009). Wellbeing predicts longevity among healthy populations and the observed positive effect is for example of similar magnitude as the negative effect of smoking (Steptoe and Wardle 2012; Veenhoven 2008). The World Health Organization (WHO) has recommended that national mental health policies should not only be concerned with mental disorders, but should actively promote mental health and resilience. In addition, data on wellbeing, collected in large-scale panel studies such as the British Household Panel Study (BHPS), the German Socio-Economic Panel (SOEP), or the Eurobarometer, are already used in conjunction with economic data to guide public policy. A reflection of the rising interest is the recent United Nations high-level meeting on wellbeing and happiness (April 2012), the World Happiness Report 2013 (Helliwell et al. 2013), and the OECD Guidelines on Measuring Subjective Well-being (OECD 2013), with the intention to harmonize and structure the world-wide measurement of wellbeing.

### What is wellbeing?

In general, wellbeing is conceptualized to include a continuous spectrum of positive feelings and subjective life assessments. Wellbeing conveys information regarding a broad range of behaviors and health, including physical and mental health, social relationships, leisure, and subjective states such as emotions and mental engagement.

Different definitions of wellbeing have been launched over the years. The recent OECD report (p. 10) defines subjective wellbeing as *good mental states, including all of the various evaluations, positive and negative, that people make of their lives, and the affective reactions of people to their experiences*, while the World Happiness Report, as the name reflects, focuses more on happiness, and explains that happiness is part of wellbeing. From a more philosophic point of view, wellbeing is sometimes distinguished in two basic forms: a “hedonic” form representing the sum of an individual’s positive affective experiences (Ryff et al. 2004), and a deeper “eudaimonic” form that results from striving toward meaning and a noble purpose beyond simple self-gratification (Ryan and Deci 2001). Wellbeing has also been defined as the total sum of a cognitive and an emotional or affect component (Andrews and McKennell 1980; Galinha and Pais-Ribeiro 2011). This is in line with the description given by Diener et al. (1999) that explains wellbeing as a broad category of phenomena that includes people’s emotional responses, domain satisfaction, and global judgments of life satisfaction.

Often terms like wellbeing, satisfaction with life, happiness, or quality of life are used interchangeably (Layard 2010). In practice, focus in wellbeing research is mostly on an overall measure of wellbeing, or short measures for quality of life or satisfaction with life. For example, quality of life is used to assess wellbeing in large-scale world-wide investigations in different countries, such as the Health Behavior in School aged Children study (Currie et al. 2012) and the large scale UNICEF study (2013), while most large scale panels studies use (single item) satisfaction with life questions. Happiness is much less used as an independent measure.

There is a body of evidence on the empirical association between different aspects of wellbeing. Correlations in the range of .5–.6 are reported for the association of overall wellbeing with satisfaction with life or happiness/positive affect or across dimensions (Bartels and Boomsma 2009; Diener et al. 2009). Eudaimonic and hedonic wellbeing have also been found to be highly correlated (*r* = 0.70) and reciprocally influence one another (Keyes et al. 2002; Waterman 1993). In addition, it has been found that the clustering of wellbeing dimensions is explained by one underlying common genetic effect (Bartels and Boomsma 2009).

Like all self-reported measures, survey-based measures of subjective wellbeing, are sensitive to measurement methodology, but reliability of subjective wellbeing measures have been found to be moderate to good. In a meta-analysis of multiple items life satisfaction measures Cronbachs alpha’s between .80 and .96 are reported (Diener et al. 2012) and test–retest scores are in the range of .24 (over 16 years), to .54 (over 4 years) to .84 for a period of 2 weeks to 1 month (Fujita and Diener 2005; Krueger and Schkade 2008). For single item measures test–retest correlations between .5 and .7 have been reported for time periods of 1 day to a 2-year period (Krueger and Schkade 2008; Michalos and Kahlke 2010), indicating that single item measures also perform rather well.

### The current study

Previous twin-family studies have revealed that individual differences in wellbeing and its components satisfaction with life, happiness, and quality of life, are accounted for by both genetic as well as environmental factors but the range in estimates is large. Here, the twin-family studies on wellbeing, satisfaction with life, happiness, and quality of life were reviewed. Subsequently, two meta-analyses (one for wellbeing and one for satisfaction with life) were carried out to provide a more robust estimate of the heritability of wellbeing and satisfaction with life. For the wellbeing meta-analysis the largest set of independent studies with *any* wellbeing measure was brought together. For the satisfaction with life meta-analysis, independent study selection focussed on studies with a life satisfaction measure. Due to the limited amount of independent studies for happiness and quality of life no separate meta-analyses were conducted for these constructs.

## Methods

### Literature search and study inclusion criteria

To collect studies on wellbeing and its components a search of the electronic databases PubMed (http://www.ncbi.nlm.nih.gov/pubmed) and ISI web of knowledge (http://apps.webofknowledge.com/) was conducted using the following keywords: *wellbeing/happiness/satisfaction with life AND twin/twins/heritability/genes*. No filter regarding date range or age range was specified. Animal studies and studies published in foreign languages were excluded. This search identified 165 unique papers. Abstracts of the remaining papers were examined. Only papers containing information relevant to the heritability of happiness, satisfaction with life, or wellbeing were included, resulting in 24 papers that were considered to be included for the current review. Based on the reference list of these papers, and inspection of possible missing publications by the main authors of the identified papers, 6 additional publications were identified. Presentation of the meta-analysis results at the 44th Behavior Genetic Association Meeting (2014, Charlottesville, VA, USA) resulted in an offer to include a large Finnish dataset (Koivumaa-Honkanen et al. 2005). Table 1 provides an overview and description of the 30 relevant papers and the extra dataset from Finland.

**Table 1:** Overview of heritability studies into wellbeing and its components satisfaction with life, happiness, and quality of life

No.	Reference	Measure	Cohort	Age	Sex	MZ pairs (twins)	DZ pairs (twins)	rMZ	rDZ	A (95 %CI)	C (95 %CI)	E (95 %CI)	D (95 %CI)
*Wellbeing*													
**WB1**	Tellegen et al. (1988)	Well-being Scale of MPQ	MTR	31 (9)	M/F	217 MZT 44 MZA	114 DZT 27 DZA	.58 .48	.23 .18	37	10	31	22
WB2	Lykken and Tellegen al. (1996)	Well-being Scale of MPQ	MTR	20	M/F	647 MZT 75 MZA	733 DZT 36 DZA	.44 .52	.08 –.02				
WB3	Røysamb et al. (2002)	Short version of the Subjective Well-being Scale	NIPHTP	18–25	M F OS	414 527	387 441 793	.46 .53	.22 .23 .15	46 54		54 46	
WB4	Røysamb et al. (2003)	Short version of the Subjective Well-being Scale	NIPHTP	18–31	M F OS	526 777	397 655 979	.44 .41	.24 .30 .12	44 (38–50) 44 (39–50)	13 13	43 43	
WB5	Nes et al. (2005)	Short version of the Subjective Well-being Scale	NIPHTP	25.52 (3.7)	M F OS	714 936	671 862 1528*	.50 .42	.26 .28 .16	45 (41–50)		55 (50–59)	
WB6^a^	Nes et al. (2006)	Short version of the Subjective Well-being Scale	NIPHTP	21.73 (2.23)	M F	5776 twins		.49 .55	.28 .23 .18	49 (42–55) 56 (50–61)		51 (45–58) 44 (39–50)	
**WB6** ^**b**^				25.59 (3.67)	M F	7947 twins		.48 .40	.28 .25 .12	51 (44–57) 42 (36–47)		49 (43–56) 58 (53–63)	
**WB7**	Weiss et al. (2008)	Subjective Wellbeing (telephonic interview)	MIDUS	44.9 (12.1)		347	543	.37	.10	23 (7–40)		77	
WB8^a^	Keyes et al. (2010)	Emotional Wellbeing	MIDUS	44.6	M F	163 186	123 198			49.5		50.5	
WB8^b^		Psychological Wellbeing								52.3		46.7	
WB8^c^		Social Wellbeing								45.6		55.4	
WB9	Nes et al. (2010a)	Subjective Well-being	NIPHTP HUNT	25.59 (3.67) 46.40	M F OS M–S M–D F–S F–D S–S B–B B–S S	526 392	788 638 966 7924 8461 5727 6139 3041 3016 5592 13235	.45 .40	.21 .25 .13 .13 .15 .11 .14 .20 .14 .14 .26	17 27		64 65	19 8
WB10	Nes et al. (2010b)	Subjective Well-being	NIPHTP	19–31	Ms Fs Mm Fm Md Fd	243 248 129 278 136 216	161 159 97 235 119 210	.49 .55 .41 .33 .49 .24	.20 .27 .17 .22 .26 .23	51 (42–59) 54 (46–61) 41 (30–52) 39 (29–48)		49 46 59 51**	
WB11^a^	Kendler et al. (2011a)	Emotional Wellbeing	MIDUS	44.6 (12.2)	M_95 F_95	163 186	123 198			49 (34–61)		51 (40–67)	
				53.9 (11.8)	M_05 F_05	112 128	79 134			40 (27–57)		60 (44–74)	
WB11^b^		Social Wellbeing								45 (32–57)		55 (44–70)	
WB11^c^		Psychological Wellbeing								54 (43–67) 51 (43–64)		46 (33–61) 49 (38–61)	
WB12^a^	Kendler et al. (2011b)	Emotional Wellbeing	MIDUS	44.6 (12.2)	M_95 F_95	163 186	123 198			48		52	
WB12^b^		Social Wellbeing								45		55	
WB12^c^		Psychological Wellbeing								55		45	
WB13	Bartels et al. (2012)	Wellbeing Factor score	NTR	16.41 (1.56)	M F OS B S	528 778	455 559 1085 519 (ind) 661 (ind)	.41 .47	.11 .24 .20				
**WB14**	Franz et al. (2012)	Well-being Scale of MPQ	VETSA	55.4 (2.47)	M	336	277	.38	.12	35 (18–45)	2 (0–15)	63 (55–72)	
**WB15**	Bartels et al. (2013)	Wellbeing Factor score	NTR	16.41 (1.56)	M F OS sibs	551 792	476 571 1121 1474 (ind)	.33 .45	.20 .29 .20	34 (28–39) 47 (42–51)		66 (61–72) 53 (49–58)	
*Satisfaction with life*													
**LS1**	Bergeman et al. (1991)	The Life Satisfaction Index Z (LSI-Z)	STR/SATSA	65.6 (8.2)	M/F	95 MZT 64 MZA	133 DZT 132 DZA			25 (15–33)		75	
LS2^a^	Harris et al. (1992)	The Life Satisfaction Index Z (LSI-Z)	STR/SATSA	50.8 (10.4)	M/F	95 MZT 48 MZA	108 DZT 131 DZA	.33 .18	.27 .15			100	
LS2^b^				72 (4.7)	M/F	30 MZT 33 MZA	73 DZT 46 DZA	.49 .36	.19 .29	48		52	
**LS3**	Stubbe et al. (2005)	The Satisfaction With Life Scale^4,5^	NTR	33.2 (11.3)	M F OS	235 (647) 611 (1572)	88 (345) 282 (822) 276 (943) 1455 siblings	.31 .40	−.01 .10 .11	0 (0–16)		62 (56–67)	38 (20–44)
**LS4**	Koivumaa-Honkanen et al. (2005)	Life Satisfaction (4 items)	FTC	35 (18–95)	M/F	3731	8135	.30	.15	30 (24–32)		70 (68–72)	
LS5	Johnson et al. (2006)	Self-composed life satisfaction scale based on 3 items	MIDUS	25–74	M F	172 195	138 214			24 (0–48)	11 (0–51)	65 (34–100)	
LS6	Nes et al. (2008)	Life Satisfaction (single item)	NIPHTP	18–31	M F OS	511 756	374 605 917	.35 .32	.12 .18 .10	35 (26–42) 18 (4–31)	– 11 (1–24)	65 (58–74) 71 (66–76)	
LS7	Bartels et al. (2009)	Satisfaction with life Scale	NTR	14–25	M F OS	321 449	264 326 503 972 siblings	.44 .48	.08 .22 .17	9 (0–13)		53 (48–58)	38 (17–50)
LS8	Franz et al. (2012)	Life Satisfaction (1 item)	VETSA	55.4 (2.47)	M	336	277	.22	.06	19 (7–28)	2 (0–13)	79 (70–88)	
LS9	De Neve et al. (2012)	Life Satisfaction (1 item)	Add Health	14–19	M/F	217	219	.33	.13	33 (25–41)		67 (61–73)	
**LS10**	Hahn et al. (2013)	Life Satisfaction	(GSOEP) + additional twins	40.2	M/F SIB MoCh GpaCh	202	147 419 438 102	.48	.08 .45 .49	14 17	6 32	64 31	16 20
LS11	Nes et al. (2013)	Life Satisfaction (1 item)	NIPHTP	21.7 (18–25)		1680 pairs		.43 .38	.25 .20 .10	40 (27–51) 32 (20–44)		60 (50–73) 68 (57–80)	
*Happiness*													
HAP1	Schnittker (2008)	Happiness	MIDUS	25–74	M/F	477	317	.43	.21	36	6	58	
HAP2	Bartels and Boomsma (2009)	Subjective Happiness Scale^6^	NTR	14–25	M F OS	321 449	264 326 503 972 siblings	.31 .46	.08 .17 .15	14 (13–27)		60 (59–66)	26 (11–32)
HAP3^a^	Bartels et al. (2010)	Subjective Happiness Scale^6^	NTR	17	M F OS	386 545	299 381 641 1112 siblings	.19 .42	.08 .17 .18	22 (16–28) 41 (37–45)		78 (72–84) 59 (55–63)	
HAP3^b^				33	M F OS	241 636	121 317 310 907 siblings	.29 .43	.10 .17 .13	22 (16–28) 41 (37–45)		78 (72–84) 59 (55–63)	
*Quality of life*													
QOL1	Bartels and Boomsma (2009)	Quality of Life in general	NTR	14–25	M F OS	321 449	264 326 503 972 siblings	.42 .53	.10 .26 .16	22 (6–24)		53 (52–57)	25 (23–25)
QOL2	Bartels et al. (2009)	Quality of life at present	NTR	14–25	M F OS	321 449	264 326 503 972 siblings	.40 .32	.09 .15 .21	35 (22–41)		64 (59–69)	01 (0–1)
QOL3	van der Aa et al. (2010a)	Quality of Life in general	NTR	13–20	M	290	232	.38 (ND)	.20 (ND)	30 (18–37)	0 (0–9)	70 (63–77)	
								.23 (D)	.14 (D)	30 (18–37)	0 (0–9)	70 (63–77)	
					F	432	309	.46 (ND)	.36 (ND)	43 (25–52)	3 (0–18)	54 (48–60)	
								.35 (D)	.10 (D)	42 (00–58)	1 (0–38)	56 (42–75)	
					OS		566		.24 (ND)				
									.11 (D)				
					B–B		1000 sibs		.04 (ND)				
									.02 (D)				
					S–S				.18 (ND)				
									.25 (D)				
					B–S				.19 (ND)				
									.11 (D)				
*The Ryff’s Scales*													
**R1** ^**a**^	Gigantesco et al. (2011)	Ryff’s—Autonomy	ITR	23–24	M/F/OS	65/72	39/58 /50	.45	.09	41 (27–53)		59 (47–73)	
**R1** ^**b**^		Ryff’s—Environmental Mastery						.63	.26	62 (51–71)		38 (29–49)	
**R1** ^**c**^		Ryff’s—Personal Growth						.33	.23	37 (24–49)		63 (51–76)	
**R1** ^**d**^		Ryff’s –Positive Relations						.65	.26	64 (53–72)		36 (28–47)	
**R1** ^**e**^		Ryff’s—Purpose in Life						.46	.21	47 (35–58)		53 (42–65)	
**R1** ^**f**^		Ryff’s—Acceptance						.58	.25	58 (47–67)		42 (33–53)	
R2	Franz et al. (2012)	Ryff’s Psychological Well-Being Scale	VETSA	55.4 (2.47)	M	336	277	.51	.21	50 (34–58)	1 (0–14)	49 (42–57)	
R3	Kubarych et al. (2012)	Ryff’s Psychological Well-Being Scale	VETSA	55.8 (2.6)	M	110	92			47 (32–59)		53 (41–68)	
R4^a^	Archontaki et al.,^2013^	Ryff’s—Autonomy	MIDUS	45 (12)	M/F OS	240	357 240	.41	.04	36 (18–57)		64 (54–76)	
R4^b^		Ryff’s—Mastery						.35	.10	32 (24–43)		68 (57–79)	
R4^c^		Ryff’s—Personal Growth						.38	.22	38 (21–57)		62 (50–76)	
R4^d^		Ryff’s—Positive Relations						.38	.12	36 (24–51)		64 (52–77)	
R4^e^		Ryff’s—Purpose in Life						.30	.15	30 (20-43)		70 (59–80)	
R4^f^		Ryff’s—Acceptance						.47	.14	39 (30-46)		61 (53–69)	
R4^g^		Ryff’s—Autonomy						.41	.04	36 (18–57)		64 (54–76)	

The studies that were selected for the meta-analysis of wellbeing are bold

The studies that were selected for the meta-analysis of Satisfaction with Life are bold underlined

*ND* not divorced, *D* divorced, *MoCh* mother–child, *GpaCh* grandparent–child, *Add Health* The National Longitudinal Study of Adolescent Health, *FTC* Finnish Twin Cohort, *GSOEP* German Socioeconomic Panel Study, *HUNT* The Nord-Trøndelag Health Study, *ITR* Italian Twin Registry, *MIDUS* Midlife Development in the United States, *MTR* Minnesota Twin Registry, *NTR* Netherlands Twin Register, *NIPHTP* The Norwegian Institute of Public Health Twin Panel, *STR/SATSA* Swedish Twin Registry Swedish Adoption/Twin Study of Aging (SATSA), *VETSA* Vietnam Era Twin Study of Aging, *M–S* mother–son, *M–D* mother–daughter, *F–S* father–son, *F–D* father–daughter, *S–S* sisters, *B–B* brothers, *B–S* brother–sister, *S* spouses, *Ms* concordant single males, *Fs* concordant single females, *Mm* concordant married males, *Fm* concordant married females, *Md* discordant males, *Fd* discordant females

* Sample sizes are inconsistent in Nes et al. (2005) (pairs and individuals are mixed up)

** Table 3 in Nes et al. (2010b) this estimate is given as .51, but this does not add to 1.0 given the additive genetic effects

^a, b, c, d, e, f, g^ refer to multiple results within one study

### Meta-analysis

#### Study inclusion

Two meta-analyses on twin (family) studies were conducted. For the meta-analyses only studies using independent samples could be used. First, a meta-analysis for wellbeing was conducted including all independent studies with *any* wellbeing measure or *any* measure of a wellbeing construct. Independency is achieved by selecting the most informative paper from the set of papers that derived from (partially) overlapping datasets, based on the following criteria: largest sample, sex-specific estimates, and/or reporting of confidence intervals. So for example the study of Nes et al. (2006) was chosen from all studies of the Norwegian Institute of Public Health Twin Panel (NIPHTP) and the study of Bartels et al. (2013) was chosen from the Netherlands Twin Register (NTR) studies since both of these studies are based on large samples and report sex-specific heritability estimates. The Midlife Development in the United States (MIDUS) data are used by researchers from different institutes (Archontaki et al. 2013; Johnson and Krueger 2006; Kendler et al. 2011a, b; Keyes et al. 2010; Weiss et al. 2008) and the MIDUS study collected data on wellbeing and satisfaction with life in different ways (e.g. telephonic interview, self-administered questionnaires). In the current meta-analysis the study of Weiss et al. (2008) is chosen as the independent MIDUS sample since, given the inclusion criteria, this studied covered the largest sample size. Finally, one study (Gigantesco et al. 2011) based on the Ryff’s Psychological Well-Being Scale Revisited (Ryff and Keyes 1995) was included in the meta-analysis, since this study was based on an independent sample with *any* wellbeing measure. To obtain one overall heritability estimate for this study to be included in the meta-analysis the estimates of the specific subscales were averaged.

For the meta-analysis on satisfaction with life, only independent studies with a satisfaction with life measure were included. For samples with multiple estimates the same inclusion criteria as used for the wellbeing meta-analysis were followed. Within the satisfaction of life meta-analysis two studies of the Netherlands Twin Register are included, since these two studies are based on completely independent dataset. The paper by Stubbe et al. (2005) is based on data of the Adult Netherlands Twin Register (Willemsen et al. 2013), while the paper of Bartels and Boomsma (2009) is based on data of the Young Netherlands Twin Register (van Beijsterveldt et al. 2013).

### Analysis

The broad heritability estimates (additive + non-additive) of the selected studies were meta-analyzed by computing the weighted average heritability (Li et al. 2003). To this end the heritability estimates from the independent studies were weighted by the number of participants in the study. Whenever different estimates for males and females were reported, the sex-specific estimates were treated as belonging to independent studies. For studies without sex-difference in heritability the equated estimate was used. Some samples did not report confidence intervals. In these cases, the confidence intervals (CIs) were estimated based on the reported CIs of the other studies. With these CIs the weighted mean standard deviation was calculated, which was used to calculate the stander error and thus the CI of the studies for whom CIs were lacking (Li et al. 2003). Calculations were conducted in Excel for Mac 2011 (Version 14.3.9).

## Results

### Literature review

All studies that were identified following the search and selection criteria as described in the method section are presented in Table 1. Studies are sorted by phenotype, either overall wellbeing or one of its components. For each study the literature reference, wellbeing measure, name of the study cohort, age of the sample, gender of the sample, sample size (per zygosity if provided), twin (-family) correlations, and estimates of standardized variance components are provided. Confidence intervals are included in the table when reported in the paper. The results of one study in Table 1 (LS4) are based on analysis run by the author of this manuscript. The data are described in Koivumaa-Honkanen et al. (2005) and data were used in a standard variance–covariance structural equation modeling frame-work to obtain heritability estimates and confidence intervals.

### Description of study designs and samples

The majority of the studies applied the classical twin design with a comparison of monozygotic and dizygotic twin covariance/correlation with both same-sex as well as opposite sex twin pairs. Two studies (Franz et al. 2012; Kubarych et al. 2012) included men only by design since they made use of the data of the Vietnam Era Twin Study of Aging. Five studies (De Neve et al. 2012; Johnson and Krueger 2006; Kendler et al. 2011a, b; Keyes et al. 2010) choose to include same-sex twins only and one study (Schnittker 2008) only refers to MZ and DZ twins. Four studies (Bergeman et al. 1991; Harris et al. 1992; Lykken and Tellegen 1996; Tellegen et al. 1988) included reared apart twins pairs besides twins reared together. Eight studies applied an extended twin design. Six of these eight studies used data of additional non-twin siblings (Bartels and Boomsma 2009; Bartels et al. 2010, 2012, 2013; Stubbe et al. 2005; van der Aa et al. 2010a), one study combined data from a twin cohort with data of a population based register of nuclear families, providing information on sibling dyads (Nes et al. 2010a) and one combined a twin sample with a national panel study (Hahn et al. 2013). Mean age of study participants in the studies presented in Table 1 ranges from 14 to 72, with the majority of the studies focusing on middle adulthood. Only two studies (Bergeman et al. 1991; Harris et al. 1992) included elderly (65+) individuals, while 6 studies (Bartels and Boomsma 2009; Bartels et al. 2010, 2012, 2013; De Neve et al. 2012; van der Aa et al. 2010a) focused on wellbeing from early adolescence to young adulthood. Most studies take age into account at the mean level but the effect of age on the variance components has not systematically been studied. In addition, only three studies (Kendler et al. 2011b; Lykken and Tellegen 1996; Nes et al. 2006) applied a longitudinal study design and found that genetic factors are the major source for stability in wellbeing over time. The Finnish data (Koivumaa-Honkanen et al. 2005), used to estimate the heritability estimate, are a combination of two time points (data collected in 1975 and 1981), in which the first measure is taken first and the second measure is taken when the first was missing. Finally, two studies (Bartels et al. 2010; Harris et al. 1992) investigated cohort effects by splitting the sample in two age groups. Bartels and colleagues did not find a difference in genetic architecture between adolescents (mean age 17) and adults (mean age 33), while Harris and colleagues report a remarkable absence of genetic influences in late adulthood (mean age 50.8) and a heritability of 48 % in elderly (mean age 72).

### Wellbeing measures

A wide variety of wellbeing measures has been used, some covering specific components of wellbeing, such as satisfaction with life or happiness, while others capture the overall wellbeing construct. An overview of measures, including available information on number of items and reliability, used in the reviewed studies is provided in Table 2. Obviously, certain cohorts implemented particular measures in their protocol, which have subsequently been used in multiple studies. Cohorts that used an overall wellbeing measure are the Minnesota Twin Registry, using the Well-being Scale of the Multidimensional Personality Questionnaire (MPQ; (Tellegen 1982)) and the Norwegian Institute of Public Health Twin Panel using a short version of the Subjective Well-being Scale (Moum et al. 1990). The Netherlands Twin Register adopted three wellbeing measure in their longitudinal cohort study (the Satisfaction With Life Scale (Diener et al. 1985), the Subjective Happiness Scale (Lyubomirski and Lepper 1999), and the Cantril Self-Anchoring Striving Scale (Cantril 1965). In a multivariate study they found that the correlations between these measures was moderate to high and that all measures loaded on one underlying genetic factor (Bartels and Boomsma 2009). Ever since they either used the separate measures or a weighted factor score representing wellbeing. The Swedish Twin Registry (including the Swedish Adoption/Twin Study of Aging (SATSA)) applied the Life Satisfaction Index Z (LSI-Z) (Wood et al. 1969). Several different measures of wellbeing have been collected by telephone or survey in the MIDUS (Midlife Development in the United States) panel study and these data are used in various ways. Both Johnson and Krueger (2006) and Weiss et al. (2008) composed a life satisfaction/wellbeing scale based on 3 items, while Schnittker (2008) focused on happiness based on six ‘happiness’ items from a variety of previously validated instruments. In addition, 3 measures of mental wellbeing (emotional, psychological, and social) have been developed by Keyes (1998) applying factor analysis on several wellbeing measures in MIDUS. Finally, the Ryff’s Scales of Psychological Well-being (Ryff and Keyes 1995) have been collected in the MIDUS sample. The Vietnam Era Twin Study of Aging (VETSA) applied the Ryff’s Psychological Well-being Scale, the Well-being scale of the MPQ, and an item on satisfaction with life. The Italian Twin Register used the Ryff’s Scales of Psychological Well-being with three items per dimension and the Add Health study used one item to assess Satisfaction with Life. The German Socioeconomic Panel study and the extra collected German twin samples used a life satisfaction factor score based on 5 items.

**Table 2:** Measurement instruments used to assess wellbeing and its components within the behavior genetics literature

Instrument	Reference	Study in which it is used (see Table 1)	Subscale	*N* of items	Items	Response scale	α
*WELLBEING*							
Multidimensional Personality Questionnaire (MPQ)	Tellegen (1982, Tellegen 1985), Tellegen and Waller (1994)	WB1, WB2	Well-being Scale	23	e.g. ‘Does fun things’ ‘Has a happy disposition’ ‘Has interesting experiences’ ‘Optimistic, hopeful’		.92
Subjective Wellbeing Scale-Short version	Moum et al. (1990)	WB3, WB4, WB5, WB6, WB9, WB10	SWB-index	4 (WB3: sum score) (WB4,WB5, WB6: weighted mean score index)	1. ‘When you think about your life at present, would you say you are mostly satisfied with your life, or mostly dissatisfied?’	6 point scale *1 extremely satisfied* *6 very dissatisfied*	.71
					2. ‘Are you usually happy or dejected?’	5 point scale *1 dejected* *5 happy*	
					3. ‘Do you mostly feel strong and fit or tired and worn out?’	4 point scale *1 very strong* *4 tired and worn out*	
					4. ‘Over the last month, have you suffered from nervousness?’	4 point scale *1 almost all the time* *4 never*	
Subjective Well-being	Diener et al. (1999)	WB7	–	3 items (sumscore)	Particpants were asked in a telephonic interview (1) how satisfied participants were with their life (2) how much control subjects felt they had over their lives (3) how satisfied they were with their life overall	4 point scale	–
Emotional Well-being	Bradburn (1969)	WB8, WB11, WB12	–	6 items on positive affect (sumscore)	How much of the time during the past 30 days they felt (1) cheerfull, (2)in good spirit, (3)extreme happy, (4)calm and peaceful, (5)satisfied and (6)full of life	5 point scale *1 none of the time* *5 all of the time*	.88
Psychological Well-being	Ryff and Keyes (1995)	WB8, WB11, WB12	–	6 scales of 3 items each (sumscore)	How well each item described how they generally functioned (1) Self-acceptance (2) Positive relationships with others (3) Personal growth (4) Purpose in life (5) Environmental Mastery (6) Autonomy	7 point scale *1 strongly agreed* *7 strongly disagreed*	.76
Social Well-being	Keyes (1998)	WB8, WB11, WB12		5 scales of 3 items each (sumscore)	How well each item described how they generally functioned (1) Social acceptance (2) Social growth (3) Social contribution (4) Social coherence (5) Social integration	7 point scale *1 strongly agreed* *7 strongly disagreed*	.72
Multidimensional Personality Questionnaire (MPQ)-NZ	Patrick et al. (2002)	WB14	Well-being Scale	11	e.g. I often feel happy and satisfied for no particular reason		.80
*Satisfaction with life*							
Life Satisfaction Inventory-Z (LSI-Z)	Wood et al. (1969)	LS1, LS2	–	13 (sumscore)	e.g. ‘As I grow older, things seem better than I thought they would be’ ‘These are the best years of my life’	5 point scale 1 *strongly agree* 5 *strongly disagree*	.81
The Satisfaction With Life Scale (SWLS)	Diener et al. (1985)	LS3, LS7, WB13, WB15	–	5 (sumscore)	e.g. ‘In most ways my life is close to my ideal’	7 point scale 1 *strongly disagree* 7 *strongly agree*	.86
Single item measuring life satisfaction		LS6, LS11	–	1	‘When you think about your life at present, would you say that you are mostly satisfied with your life, or mostly dissatisfied?’	6 point scale *0 extremely satisfied* *5 very dissatisfied*	.65–.69
Single item measuring life satisfaction		LS9		1	‘How satisfied are you with your life as a whole?’	5 point scale 1 *very dissatisfied* 5 *very satisfied*	–
Life satisfaction	Allardt (1973)	LS4		4 (sumscore)	subjects were asked to rate: interest in life happiness ease of living loneliness	4 point scale 1 *very interesting/happy/easy/not at all lonely* 2 *fairly interesting/happy, easy* 4 *fairly boring/unhappy/* *hard/lonely* 5 *very boring/unhappy/* *hard/lonely*	
Life satisfaction	Cummings (2000)	LS5		3 (sumscore)	Participants were asked how satisfied they were with their lives, how satisfied they were with themselves, and the degree to which they felt their lives the best possible overall	6 point scale	.62
Life satisfaction		LS10		5 items (factorscore)	household income personal income health housing leisure	11-point scale 0 *completely dissatisfied* 10 *completely satisfied*	.71
*Happiness*							
Happiness		HAP1	–	6 items (mean score)	‘During the past 30 days, how much of the time did you feel: cheerful? In good spirits? Extremely happy? Calm and peaceful? Satisfied? Full of life?	5 point scale *1 all of the time* *5 none of the time*	.91
Subjective Happiness Scale	Lyubomirski and Lepper (1999)	HAP2, HAP3, WB13, WB15	–	4 (sumscore)	e.g. ‘On the whole I’m a happy person’ ‘On the whole, I’m not very happy’	7 point scale *1 strongly disagree* *7 strongly agree*	.86
*Quality of life*							
The Cantril Self-Anchoring Striving Scale	Cantril (1965)	QOL1, QOL3, WB11, WB12, WB13, LS8, WB15	–	1	Please imagine a ladder with steps numbered from zero at the bottom to 10 at the top The top of the ladder represents the best possible life for you and the bottom of the ladder represents the worst possible life for you On which step of the ladder would you say you personally feel you stand		.41 (elderly) .32 (students)
Adjusted version of The Cantril Self-Anchoring Striving Scale		QOL2		1	Please imagine a ladder with steps numbered from zero at the bottom to 10 at the top The top of the ladder represents the best possible life for you and the bottom of the ladder represents the worst possible life for you How do you feel at the moment?		
Ryff’s Scales of Psychological Well-Being	Ryff et al. (2007)	R4	Autonomy, Environmental Mastery, Personal Growth, Positive Relations, Purpose in Life, Self-Acceptance	42 items sumscore per dimension		7 point scale 1 *strongly agree* 7 *strongly disagree*	.70–.84
Ryff’s Psychological Well-Being Scale-Revisited	Ryff and Keyes (1995)	WB14, R1, R2, R3	Self-Acceptance Environmental Mastery Positive Relations Purpose in Life Personal Growth Autonomy Total Score	18 items (6 dimensions with 3 items each)		6 point scale 1 *strongly disagree* 6 *strongly agree*	.52 .49 .56 .33 .40 .37 .80*

* Based on VETSA data

### Heritability estimates

Altogether the studies in Table 1 provide us with 70 heritability estimates (some studies provide multiple estimates either based on multiple measures, multiple time points, or separate estimates for males and females) ranging from 0 to 64 %.

Heritability estimates for overall wellbeing range from 17 to 56 %. For the components of wellbeing the ranges are 0–60 % for satisfaction with life, 22–41 % for happiness, and 22–42 % for quality of life, respectively. One study (Harris et al. 1992) reported that all variance in satisfaction with life was due to nonshared environmental factors.

The majority of the studies conducted classic heritability estimation based on variance–covariance matrices of MZ and DZ twins or in some cases extended designs. The exceptions are Tellegen et al. (1988) and Harris et al. (1992) who used the within-between method of Jinks and Fulker (1970) and Lykken and Tellegen (1996) who reported twin correlations only.

The minority of the heritability estimates were based on studies which applied a univariate model (Bartels et al. 2010; De Neve et al. 2012; Lykken and Tellegen 1996; Nes et al. 2010a, b; Røysamb et al. 2002; Stubbe et al. 2005; Tellegen et al. 1988). Other studies applied a multivariate model to multiple measures of wellbeing (Archontaki et al. 2013; Bartels and Boomsma 2009; Gigantesco et al. 2011; Keyes et al. 2010), longitudinal data (Kendler et al. 2011a, b; Koivumaa-Honkanen et al. 2005; Lykken and Tellegen 1996; Nes et al. 2006, 2013), or applied a multivariate framework to investigate the overlap of wellbeing with other phenotypes, such as somatic health variables (Harris et al. 1992; Røysamb et al. 2003), sleep (Nes et al. 2005), mental health/illness (Bartels et al. 2013; Kendler et al. 2011a, b; Nes et al. 2008, 2013), social support (Bergeman et al. 1991; Schnittker 2008), exercise behavior (Bartels et al. 2012), or personality (Hahn et al. 2013; Weiss et al. 2008). Two studies combined wellbeing with self-esteem and either mental illness (Franz et al. 2012) or hippocampal volume (Kubarych et al. 2012). Finally, three studies applied genetic moderation models to estimate the heritability under different conditions such as different financial situations and perceived control (Johnson and Krueger 2006), parental divorce (van der Aa et al. 2010a), or marital status (Nes et al. 2010b).

Five studies, all extended twin designs, report significant non-additive genetic effects (Bartels and Boomsma 2009; Hahn et al. 2013; Nes et al. 2010a; Stubbe et al. 2005; Tellegen et al. 1988), while eight studies reported evidence for shared environmental influences, although some with a zero in the confidence interval (Franz et al. 2012; Hahn et al. 2013; Johnson and Krueger 2006; Nes et al. 2008; Røysamb et al. 2003; Schnittker 2008; Tellegen et al. 1988; van der Aa et al. 2010a). The absence of significant findings for non-additive genetic or shared environmental effect by no means indicates the absence of these effect, since it could also reflect a lack of power to detect these variance components (Posthuma and Boomsma 2000). Some indirect evidence for the presence of non-additive genetic effects in wellbeing is provided by the recent finding of molecular genetic evidence for wellbeing (Rietveld et al. 2013). In this study a SNP heritability of 12–18 % is reported, reflecting additive genetic effects. This estimate is fully in line with the estimate of additive genetic influences in the above-mentioned more powerful extended twin studies. The remaining variance in the twin-sibling studies is non-additive genetic variance, indicating that part of the reported heritability estimates of the studies in Table 1 probably include both additive and non-additive genetic effects. Evidence for a possible influence of shared environment is provided by the finding of significant shared environmental influences on wellbeing and satisfaction with life in two powerful studies that combined twins and nuclear families or panel data (Hahn et al. 2013; Nes et al. 2010a).

Two cohorts (the Norwegian Institute of Public Health Twin Panel and the Netherlands Twin Register), reported significant sex-differences in heritability in some of their studies (Bartels et al. 2010, 2013; Nes et al. 2006, 2008; Røysamb et al. 2003). The NTR consistently report higher estimates for females versus males, which is also observed in most of the NIPHTP studies, except for the heritability estimates at time2 in Nes et al. (2006) and the estimates in Nes et al. (2008).

**Fig. 1:**
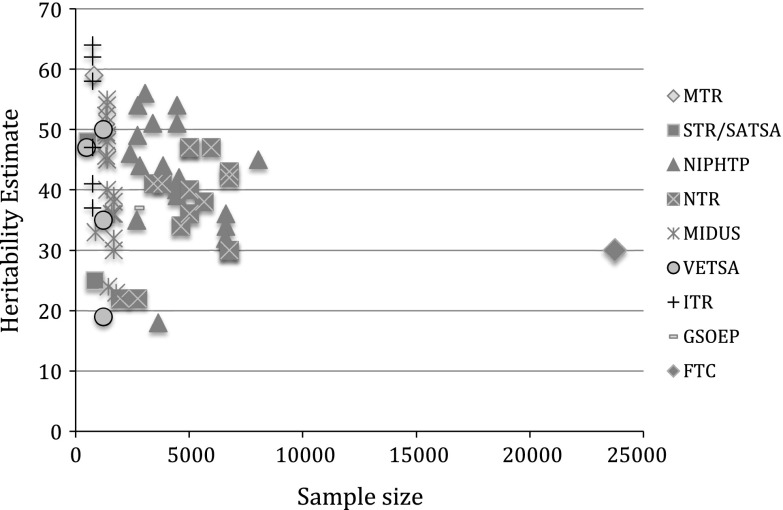
Scatterplot representing the association between variance in heritability estimate and sample size separated by cohort

Scattering out the spreading in heritability estimates versus the sample size of the studies (see Fig. 1), shows that less variance in estimates is observed with increasing sample size. In addition, larger within cohort variance in heritability estimate is observed when multiple measures of wellbeing are used (e.g. MIDUS).

Finally, the heritability studies cover a large age range. The youngest twins are 13 years of age (van der Aa et al. 2010a), while the oldest are 87 years of age (Harris et al. 1992). Comparing the heritability estimates of different studies does not reveal a large age effect, with the exception of the study by Harris et al. (1992), in which no evidence for genetic influences on satisfaction with life in late adulthood is reported.

### Meta-analyses

**Fig. 2:**
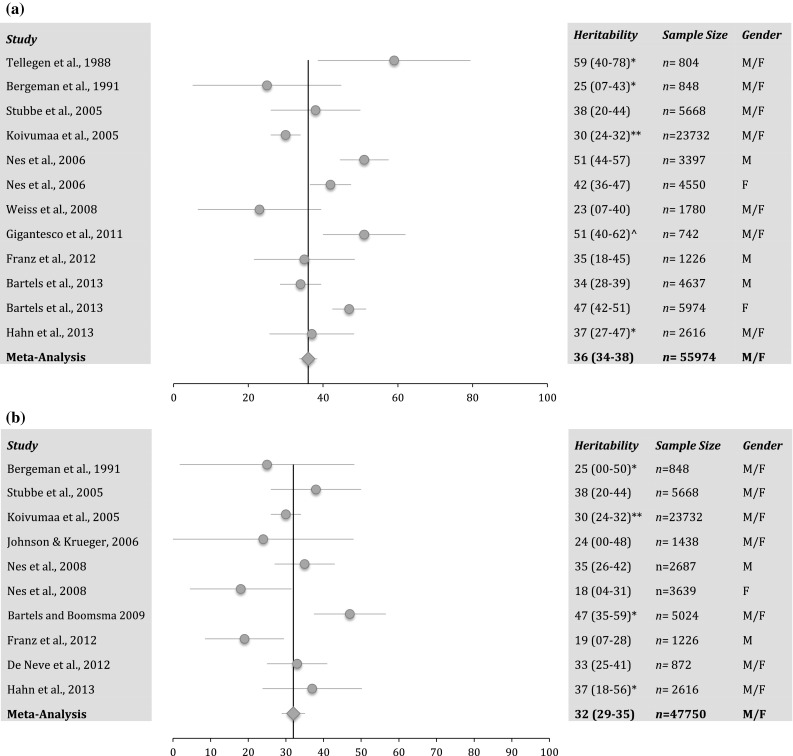
**a** Heritability estimates and 95 % confidence intervals for the studies used in the meta-analysis of wellbeing. The bottom line (Meta-Analysis) shows the weighted heritability estimate and confidence interval. *CI’s estimated based on the other studies; **heritability estimated by author after receiving data from PI of Finnish Twin Cohort; ^ the heritability is the average heritability of the 6 Ryff’s dimensions. **b** Heritability estimates and 95 % confidence intervals for the studies used in the meta-analysis of satisfaction with life. The bottom line (Meta-analysis) shows the weighted heritability estimate and confidence interval. *CI’s estimated based on the other studies; **heritability estimated by author after receiving data from PI of Finnish Twin Cohort

The studies that were selected for the meta-analysis of wellbeing (bold faced studies WB1, LS1, LS3, LS4, WB6b, WB7, R1, WB14, WB15, LS10 in Table 1) and satisfaction with life (underlined studies LS1, LS3, LS4, LS5, LS6, LS7, LS8, LS9, LS10 in Table 1) and the results of the meta-analysis are presented in Fig. 2a, b. Twelve heritability estimates from 10 studies were used for the meta-analysis of wellbeing, ranging from 23 to 59 %. The mean age of the included samples ranged from 16.4 to 65, with an average of 37. Only one study sample was under age 20 and one study population was above 65.

Ten heritability estimates, derived from 9 independent samples, were used for the meta-analysis of satisfaction with life, ranging from 18 to 47 %. The age of the included samples ranged from 14 to 74, with three studies including participant under 20 and two studies with participants over 65.

Each study is represented by a dot (point estimate) and line (95 % confidence interval). When confidence intervals were not reported in the original paper, these were estimated based on the CI’s of the other studies. The result of the meta-analysis is presented in the last line of the table. The weighted average heritability of wellbeing is 36 % (34–38), while the weighted average heritability for satisfaction with life is 32 % (29–35). The meta-analytic point estimate of wellbeing falls within the confidence intervals of 6 of the 12 (50 %) studies reflecting expected heterogeneity in a broad measure such as wellbeing. For satisfaction with life, a more homogenous picture is found, with the point estimate falling in 7 of the 10 confidence intervals (70 %).

## Discussion

The identification of multiple studies covering the genetic architecture of wellbeing within the scientific literature reflects the increasing interest in the topic. Results of these twin-family studies into the genetic and environmental influences on wellbeing show a range of heritability estimates (0 %–64 %). The two meta-analyses, one for satisfaction with life and one for wellbeing, showed that 32–36 % of the variance, respectively, is accounted for by genetic effects. These results provide a more robust estimate of the relative influence of genetic effects on wellbeing. Although such an overall weighted measure provides guidance within a rapidly growing field of interest, it should also be interpreted with care.

### Considerations and limitations

Several considerations and limitations need to be taken into account when interpreting the meta-analytic heritability estimates. *First*, there is an ongoing debate on the meaning of wellbeing and how to measure it, which is also reflected in the introduction section of the paper and in Table 2. The large variety in wellbeing questionnaires, scales, subscales and items, makes a meta-analysis vulnerable to heterogeneity. This is also reflected in the differences in heritability estimates of the different studies and the absence of overlap between the meta-analytic point estimate and the 95 % confidence intervals of some studies. Encouraging in this respect is the finding of the overlap between the meta-analytic point estimate of satisfaction with life and the confidence intervals of most of the included studies. For wellbeing the meta-analytic point estimate falls within the about half of the confidence intervals, so even for such a broad measure, making it prone to heterogeneity, the picture is far from alarming. Even more so, if one takes into account that the CIs that do not include the meta-analytic point estimates are, among others, from the two studies in which sex-specific estimates were reported (and thus used in the meta-analysis) (Bartels et al. 2013; Nes et al. 2006).

This leads to the *second* limitation of the possible sex-differences in genetic architecture of wellbeing. Two cohorts (the NIPHTP and the NTR), reported significant sex-differences in heritability in some of their studies (Bartels et al. 2010, 2013; Nes et al. 2006, 2008; Røysamb et al. 2002). The NTR consistently report higher estimates for females versus males, which is also observed in most of the NIPHTP, except for the heritability estimate at time2 in Nes et al. (2006) and the estimate in Nes et al. (2008). Consequently the meta-analyses were based on the combination of male and female data, leaving no room for sex-specific estimation. So, it remains unclear if gender really matters for the causes of individual differences in wellbeing. In addition, none of the studies, systematically tested for qualitative sex-differences, so no insight in possible effects of sex-specific genes and environmental factors is available. A mixed picture is obtained when the opposite sex-twin (OS) correlations are compared to same-sex dizygotic (SS-DZ) twin correlations (see Table 1). Some studies report lower OS correlations than SS-DZ correlations (see for example the studies based on the NIPHTP sample), while in other studies no clear difference is observed (see for example the studies based on the NTR sample).

*Third*, several studies assessed multiple wellbeing measures within the same cohort of people and for sake of independence only a couple of these studies (with no overlap in participants) could be included in the meta-analyses. The best example of this limitation is given by the different studies that make use of the MIDUS survey data. Seven of the studies presented in Table 1 make use of these data. As a result only one study could be used for the wellbeing meta-analysis (Weiss et al. 2008) and one for the satisfaction with life meta-analysis (Johnson and Krueger 2006). While selection of the two used studies is based on pre-defined criteria (e.g. sample size), it still means that for example all the relevant work of Keyes et al. (2010) and Kendler et al. (2011a, b), Schnittker (2008), and Archontaki and colleagues (Archontaki et al. 2013) is not used in the meta-analysis. The same holds for several studies based on the data of the Netherlands Twin Register (Bartels et al. 2010, 2012; van der Aa et al. 2010a), and the Norwegian Institute of Public Health Twin Panel (Nes et al. 2005, 2008, 2010a, b; Røysamb et al. 2002, 2003).

*Fourth*, meta-analytic results are constrained by the characteristics of the input studies. By combining different studies a large age range was covered and only cross-sectional results were included. Two studies tested for the difference in heritability by age and report opposite results. Harris et al. (1992) report remarkable age differences in the etiology of satisfaction with life, with no evidence for genetic influences in the age group younger than 65 year, and a heritability of 48 % in elderly (>65). More recently, Bartels et al. (2010) were allowed to constrain genetic and environmental influences on happiness to be similar for younger (aged 14–19 years) and older individuals (aged 20–88 years). The studies that have used repeated measures of wellbeing to investigate the longitudinal genetic architecture (Nes et al. 2006) or the genetic influence on the overlap of the two measurement occasions (Kendler et al. 2011b; Nes et al. 2013) report large genetic influences on the stability of wellbeing. However, the exact effect of age on the genetic architecture of wellbeing and its components has yet to be determined. Additionally, the longitudinal studies were based on only two measurement occasions providing no room for more complex longitudinal modeling. Therefore, a large-scale study into the causes of stability and change in wellbeing including data of over more than 2 measurement occasions is highly warranted. Additionally, studies disentangling genetic and environmental influences on wellbeing throughout childhood are highly warranted since differences in genetic architecture between children, adolescents, and adults have been frequently observed (Haworth et al. 2010; Huppertz et al. 2012; Nivard et al. 2014).

Furthermore, some studies were based on the classical twin design, which compares monozygotic and dizygotic twins, while other studies used extended designs, including siblings, twins reared apart, and other relatives of twins. Inclusion of these extended twin designs in the meta-analysis probably has strengthened the overall finding since the heritability estimates of the particular studies are more precise, due to increase in power for variance decomposition.

*Fifth*, all studies included in the meta-analyses are based on western European and Northern American (population) based samples. This restricts the interpretation of the result for other populations. Furthermore, most studies rely on the voluntary participation of the twins and their relatives, which imaginably can result in a bias with regard to a reduced variance in wellbeing in comparison to an ‘unselected’ sample.

*Sixth,* while existence of a dynamic interplay between genes and environment, such as gene–environment interaction, gene–environment correlation, and epigenetics, is acknowledged for complex traits, it has scarcely been studied for wellbeing. All but three studies summarized in Table 1 applied a basic model with additive effects of genes and environment to explain individual differences in wellbeing. Only Johnson and Krueger (2006), Nes et al. (2010b), and van der Aa et al. (2010a), tested whether the heritability estimates were significantly moderated by level of financial resources and perceived control, marital status, or parental divorce, respectively. Heritability is found to be higher in better financial positions (due to moderation of nonshared environment and thus a change in genetic proportion of the total variance) and at higher levels of perceived control (due to increasing genetic variance) (Johnson and Krueger 2006). Genetic influences on variation on wellbeing were shown to be significantly smaller in married $\left( {h^{ 2}_{\text{m}} = { 41}\% ;h^{ 2}_{\text{f}} = { 39}\% } \right)$ than single individuals $\left( {h^{ 2}_{\text{m}} = { 51}\% ;h^{ 2}_{\text{f}} = { 54}\% } \right)$ (Nes et al. 2010b). Finally, the unstandardized additive genetic and nonshared environmental influences on Quality of Life were shown to be increased in girls from divorced families compared to girls from nondivorced families, whereas the standardized contribution was similar, due to an overall increase in variance. No effect for boys was observed (van der Aa et al. 2010a).

### Molecular genetic studies

The robust estimate provided by this meta-analysis supports the investment to try to identify genomic regions of interest for wellbeing. This is also reinforced by a recent paper that shows the first evidence for the heritability of wellbeing derived directly from molecular genetic data (Rietveld et al. 2013). Based on a pooled sample of **≈**11,500 unrelated, comprehensively-genotyped Swedish and Dutch individuals, estimates of broad-sense heritability of 5**–**10 % for single-question survey measures of wellbeing is found. This estimate increases to 12**–**18 % after correction for measurement error in the wellbeing measures. So far, however, there have been only a few attempts to find genetic polymorphisms associated with wellbeing. One study reported an association of satisfaction with life and the VNTR polymorphism on the serotonin transporter gene (*5*-*HTTLPR*), with greater satisfaction with life for the individuals with the longer variant (De Neve 2011), but follow-up work on an augmented sample from the same data did not replicate the finding (De Neve et al. 2012). A second study reports a significant association between MAOA and happiness, but only in women, with low expression being related to greater happiness (Chen et al. 2013). In addition a small, probably underpowered, genome-wide linkage study indicated genomic regions of interest on chromosome 1 and 19 (Bartels et al. 2010). Finally, a small study analyzed leukocyte basal gene expression profiles and reported distinct gene expression profiles for hedonic (positive affect) versus eudaimonic (striving, purpose) wellbeing (Fredrickson et al. 2013), but replication is warranted, especially since distinguishing hedonic versus eudaimonic wellbeing with a self-report measure is under debate (Brown et al. 2014; Cole and Fredrickson 2013, 2014; Coyne 2013). A large (*n* > 100k) genome-wide association meta-analysis is currently underway within the Social Sciences Genetic Association Consortium (http://www.ssgac.org).

## Summary and conclusion

Overall, the results of the meta-analyses, by combining and weighting the results of all available independent twin-family studies, provide more robust estimates of the broad-sense heritability of wellbeing and satisfaction with life. The results indicate that genetic factors contribute significantly by explaining about 35 % of the variance. The significant finding of genetic influences on wellbeing, the room for environmental influences, and the absence of replicated candidate gene findings call for large-scale genome-wide molecular genetic studies and investments to unravel the dynamic interplay of genes and environment in the etiology of wellbeing.
